# Estimating Trends of Population Decline in Long-Lived Marine Species in the Mediterranean Sea Based on Fishers' Perceptions

**DOI:** 10.1371/journal.pone.0021818

**Published:** 2011-07-19

**Authors:** Francesc Maynou, Mario Sbrana, Paolo Sartor, Christos Maravelias, Stefanos Kavadas, Dimitros Damalas, Joan E. Cartes, Giacomo Osio

**Affiliations:** 1 Institut de Ciències del Mar, Consejo Superior de Investigaciones Científicas, Barcelona, Spain; 2 Consorzio per il Centro Interuniversitario di Biologia Marina ed Ecologia Applicata, Livorno, Italy; 3 Hellenic Centre for Marine Research, Anavissos, Attica, Greece; Institute of Marine Research, Norway

## Abstract

We conducted interviews of a representative sample of 106 retired fishers in Italy, Spain and Greece, asking specific questions about the trends they perceived in dolphin and shark abundances between 1940 and 1999 (in three 20 year periods) compared to the present abundance. The large marine fauna studied were not target species of the commercial fleet segment interviewed (trawl fishery). The fishers were asked to rank the perceived abundance in each period into qualitative ordinal classes based on two indicators: frequency of sightings and frequency of catches (incidental or intentional) of each taxonomic group. The statistical analysis of the survey results showed that both incidental catches and the sighting frequency of dolphins have decreased significantly over the 60+ years of the study period (except for in Greece due to the recent population increase). This shows that fishers' perceptions are in agreement with the declining population trends detected by scientists. Shark catches were also perceived to have diminished since the early 1940s for all species. Other long-lived Mediterranean marine fauna (monk seals, whales) were at very low levels in the second half of the 20^th^ century and no quantitative data could be obtained. Our study supports the results obtained in the Mediterranean and other seas that show the rapid disappearance (over a few decades) of marine fauna. We show that appropriately designed questionnaires help provide a picture of animal abundance in the past through the valuable perceptions of fishers. This information can be used to complement scientific sources or in some cases be taken as the only information source for establishing population trends in the abundance of sensitive species.

## Introduction

Several long-lived marine species occur in the Mediterranean Sea, such as cetaceans (dolphins and whales), the monk seal, marine turtles and sharks. Large long-lived marine animals are threatened by various human activities and their effects, such as pollution, disease, fishing, tourism, habitat alteration or direct persecution; however, the actual long-term trends in their abundances are hard to determine due to the difficulty of making scientific observations and the lack of appropriate long-term monitoring programmes [1] [1]–[3].

Dolphins (*Delphinus delphis*, *Tursiops truncatus* and *Stenella coeruleoalba*) are still commonly sighted in the Mediterranean Sea by fishers and nature watchers. However, their population numbers have been declining in recent times, as amply documented in the literature [3]–[4]. In the past, their extermination was actively backed by governments, as dolphins were considered to prey on the nets set for small pelagics like sardines and anchovies. For instance, the Austrian Fishermen's Association in the early 1900 gave free rifles and ammunition to eastern Adriatic fishermen in order to reduce the dolphin populations [5]. In the same area there were bounties paid to fishermen for each head of a large shark or dolphin delivered to the authorities [6]. In Italy in this period there was extensive debate on the best ways to reduce cetaceans that even had the Italian Navy carrying out killing experiments with dynamite.

Dolphins were occasionally used for human consumption [7], [personal communication by fishers]. More recently, dolphins have suffered high mortality rates due to entanglement in driftnets [1],[8], and in the early 1990s, due to an epizootic which swept through the Mediterranean [9].

Marine turtles are subject to incidental capture and mortality by fishing gears, especially longlines and driftnets [8],[10]. The main species present in the Mediterranean is the loggerhead *Caretta caretta*, which nests in the eastern Mediterranean and often becomes caught in fishing gear during its migration from nesting to feeding areas in the Mediterranean Sea and adjacent Atlantic, mainly in the southern Mediterranean [10].

Unlike dolphins and turtles, cartilaginous fish (henceforth termed “sharks”, although here we include skates and rays as well) are commercial species and by-caught in different Mediterranean fisheries, and therefore sold and consumed (*ca.* 10 000 t annually in the Mediterranean in recent years [11], although actual catches may be larger due to IUU). The diversity in terms of species and habits is much larger in sharks than in dolphins and turtles. A recent review work [11] identified 49 sharks proper, 34 batoids and 1 chimaera as confirmed cartilaginous species in the Mediterranean. These 84 species range from large pelagic sharks (Lamnidae and Carcharhinidae) to small benthic sharks (Squalidae and Scyliorhinidae) and benthic batoids (which includes rays) of different sizes and life-strategies. Like dolphins and turtles, pelagic sharks are incidentally caught by driftnets and longlines [12], while benthic sharks are mainly caught by bottom trawls [8],[11]. What makes most species of sharks particularly vulnerable is their low growth rate and low reproductive potential, and generally their low resilience to exploitation [13]. In many areas of the world a decline in shark landings has been reported [14]. In the Mediterranean, species that were formerly the object of commercial fisheries, such as *Squatina* spp. and *Mustelus* spp., have been reduced to very low abundance levels [15]. According to the conservation status listed in [11], 42 of the 50 species of sharks not considered “rare or occasional” are in the “threatened” category of the IUCN red list. However, not all sharks have been driven to low abundance levels by fishing: smaller species such as *Scyliorhinus canicula* are still abundant in the trawl by-catch.

Population declines and the extinction of marine organisms may be largely underestimated due to the difficulties involved in making scientific observations [16]–[17]. However, data sources other than scientific time-series have proven useful in providing relevant information to marine scientists in cases that are normally considered “data-poor” [18]–[19]. Some studies have used traditional (or local) ecological knowledge to reconstruct temporal population trends and discover near-extinctions of marine fauna [17], [20], [21], while studies that compare the results from scientific research with evidence based on fishers' experience have shown that both sources of knowledge give similar results and can be used to detect the essential trends [22]–[23].

In many cases, such as in our study of sensitive marine species in the Mediterranean, enlisting fishermen (as observers of the marine system) may be the only way to gather information for a scientific study or can be used to complement other sparse information sources (local archives, naturalists' diaries, old scientific explorations, etc.) [24], [25], [26]. Fishers, especially retired fishermen with professional careers often exceeding 40 years, can provide ecological knowledge on the functioning of marine systems and their resources [27]. The objective of this study was to evaluate the use of fishers' memories in reconstructing abundance indices of marine long-lived megafauna, such as dolphins, turtles and sharks.

## Methods

**Figure 1:**
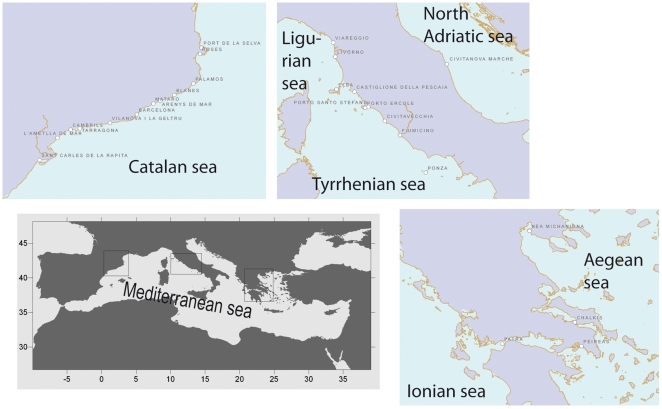
Study area. General map of the Mediterranean Sea with the port locations where the interview survey was carried out in each case study (Catalan Sea, Ligurian and Tyrrhenian Seas, North Adriatic Sea and Hellenic Seas).

The interview survey was carried out during the second semester of 2009 in ports of Catalonia (Spain), the Ligurian, Tyrrhenian and Adriatic Seas (Italy) and in ports of the Ionian and Aegean Seas (Greece) (Figure 1). In all the study areas the interview survey was restricted to retired fishermen that had worked in the trawl fishery (with the addition of small scale fishermen in the Tyrrhenian Sea case study) because the primary objective of the project within which the survey was carried out was to assess the effect of trawl fishing on Mediterranean marine habitats. The survey was conducted as part of a wider study on the historical trends of trawl fishery catches and effort (project EVOMED). The complete interview survey included questions on abundances and catches of target species, fishing techniques, vessel characteristics, catch composition and the location of fishing grounds. In the present work only the questions about dolphins, marine turtles and sharks are analyzed in detail, with qualitative comments on whales and monk seals (*Monachus monachus*). All interviews were carried out in person at the ports by experienced interviewers with a close knowledge of the fishing sector.

The interview process started with preliminary telephone contact with the Fishermen's Association in each port. This generally led to obtaining one or two addresses of local retired fishers of trawling vessels (we asked preferentially for skippers). After the interview with one person the interviewee would usually suggest further contacts with other retired fishers in the same fleet segment in the same port or neighbouring ports. It is not possible to estimate the number of existing retired fishers in each study area (sampling population) because there are no official lists. However, a rough estimate can be obtained considering that the trawl fleets consisted of 300 to 500 vessels in each study area in the early 1980s, when they were at their largest; hence, our sampling population must have been a few hundred people (100–300), taking into account deceases during the *ca.* 25 year period between the maximum activity of the trawl fishery and the time of the interviews (2009). Table 1 shows the number of fishers interviewed in each study area, their age and the year they started trawl fishing.

**Table 1:** Survey interview data showing the number of fishers interviewed in each area, their age and their experience in the fishery.

	N° of interviews	Age range of interviewees (mean)	Started fishing (range and mean)
Catalan Sea	23	49–88 (69.4)	1932–1974 (1954)
Ligurian and Tyrrhenian Seas (trawl)	35	49–85 (68.8)	1936–1982 (1958)
Ligurian and Tyrrhenian Seas (small scale fishers)	10	60–98 (74.6)	1922–1974 (1948)
North Adriatic Sea	15	64–82 (73.3)	1942–1960 (1950)
Hellenic Seas	23	45–88 (67.7)	1944–1982 (1957)
Total	106	45–98 (69.7)	1922–1982 (1955)

We show the age of the fishers at the time of the interview (2009) and the time when they started in the activity for each study area in terms of range and mean. Small-scale and trawl fishers of the Ligurian and Tyrrhenian Seas were combined for the statistical analysis.

Most fishermen, depending on their age, were only able to report on 2 time periods. The responses of each informant across different time periods were treated as random effects in the statistical model (see below) because they cannot be considered independent. However, as none of the species considered is a target of the Mediterranean trawl fishery, each interaction (sightings or catches) can be considered independent of the fishing effort in each area. For instance, the main fishing gear that negatively affects dolphins is the driftnet [1], [8], not trawl nets, but dolphins are also occasionally caught in trawl nets and commonly sighted by fishers during their work. Catches of marine turtles by trawl fishers are incidental and much less frequent than catches by driftnet fisheries [10]; however, we believe that the information on incidental catches and sightings of marine turtles reported is reliable because these large marine species are easy to recognize.

The survey covered the period from 1940 to the present divided into 3 time segments: 1940–1959, 1960–1979 and 1980–1999. For each time period, the questions related to the species of interest in the present study were: a) intentional catches of dolphins or turtles; b) sightings of dolphins, whales, seals or turtles; and c) relative catches of cartilaginous fish (“sharks”). In the questions in groups a) and b) the interviewee was asked to rate the catches or sightings as “never”, “occasional”, “frequent” and “very frequent”. The category “never” was defined as “species never seen or caught in the 20-yr period”; the category “occasional” was defined as “species seen or caught sometimes, but not regularly every year of the 20-yr period”; category “frequent” was defined as “species seen or caught regularly every year of the 20-yr period”; category “very frequent” was defined as “species seen or caught in practically every fishing trip”. For questions in group c) the choices were “less abundant” “the same”, “more abundant (twice)”, “much more abundant (three times)” than at present. The relative changes were assessed in relation to the present (loosely defined as the period 2000–2008).

Regarding the species identity within each faunal group, fishermen in general did not differentiate between the 3 species of dolphins present in the Mediterranean. Other cetaceans (whales) were very rarely caught in nets and only occasionally sighted, except in the Ligurian and Tyrrhenian Seas, where there were sufficient data for analysis. Fishers did not recognize turtles at species level either, but it is well known that *Caretta caretta* is the most frequent species by far in the area. The only species of seal in the Mediterranean is the monk seal, which is on the brink of extinction and which was extirpated from the western Mediterranean during the first half of the 20^th^ century [28]. Only the Greek fishers in our interview survey have had the chance to encounter it, but data was insufficient to attempt a statistical analysis. In terms of sharks, Mediterranean fishers do not differentiate clearly between cartilaginous fish and bony fish. Many interviewed fishers had difficulty in separating commercial sharks (such as the small spotted dog shark, *Sciliorhynus canicula*) from the general category of fish, especially in the Catalan Sea study area. Conversely, when asked about a particular species (*Mustelus*, *Squatina*), even if they had not seen one they could remember well the organism being talked about. The interviewer asked about 5 species of cartilaginous fish that had declined over the study period, and if some bony fish were spontaneously mentioned (e.g. sturgeon) the interviewer would suggest a new candidate shark species.

The statistical analysis of the responses was based on Generalized Linear Mixed Models (GLMMs) for ordinal outcomes, which is an extension of the logistic regression model to longitudinal data [29]. The response variables were naturally arranged in *C* strictly non-overlapping, ordered categories (sightings and catches of dolphins and turtles: “never”<“occasional”<“frequent”<“very frequent”; shark catches: “less abundant”<“the same”<“more abundant (twice)”<“much more abundant (three times)”) in each time period (1940–1959; 1960–1979; 1980–1999). For each dependent variable and study area, these ordinal outcomes were then regressed against a linear model containing a fixed effect (time period) and a random effect (informant). For each variable and study area, the time effect was considered significant when the full model of a significant time effect could not be rejected (log likelihood of the full model lower than log likelihood of the null model) and the slope of the time effect was significant (p<0.05, using the Z-test). Note that a decreasing trend is shown by a negative coefficient of the time variable. The routine *reoprob* of the software package STATA10 was used to compute the ordinal GLMMs ([30] provides details on the model and the software). The results are given for each study area and jointly for the entire population surveyed.

## Results

**Figure 2:**
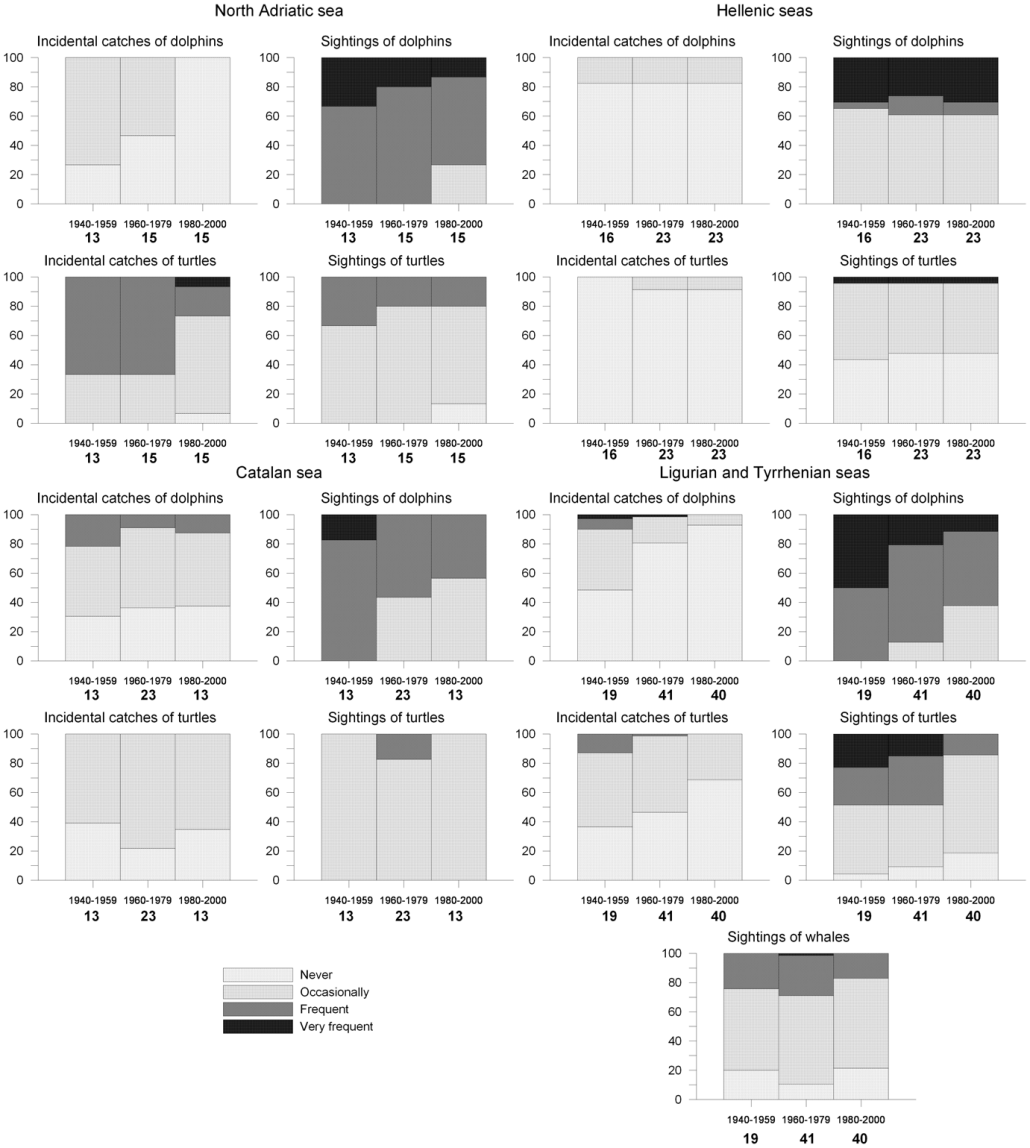
Trends in catches and sightings of large marine fauna. Frequencies in the responses to questions on catches or sightings of dolphins, sea turtles and whales in each Mediterranean case study, by time period.

Figure 2 shows the evolution of the frequency of catches and sightings of dolphins and turtles in each study area. For the Ligurian and Tyrrhenian Seas, whale (other than pilot whale) sightings are also shown. Figure 2 shows that the incidental catches of dolphins became less frequent over time in the Italian study areas (significantly decreasing time coefficient, Z-test, p<0.05 in both cases, Table 2) as well as in the Catalan Sea and have no clear trend in the Hellenic Seas (Z-test with p>0.05). Dolphin sightings, which were frequent or very frequent everywhere (except in the Hellenic Seas), have become increasingly occasional in the last study period (significantly decreasing time coefficient, Z-test, p<0.05, except in the Hellenic Seas). In addition to the frequency of sightings many fishers reported that the pods of dolphins were larger in the past and easily spotted from land; however, since the early 1960s in Catalonia and the late 1970s in western Italy only small pods (10–20 individuals) have been seen.

**Table 2:** Results of the Generalized Linear Mixed Models on ordinal outcomes in each study area.

Area	Variable tested	Null model log-likelihood	Full model log-likelihood	coefficient of time effect	Z	p(Z)
Catalan Sea	Incidental catches of dolphins	−45.420	−38.270	−1.384	−2.110	0.035
	Incidental catches of turtles	−27.870	−26.960	−1.142	−1.000	0.318
	Dolphin sightings	−29.2868	−25.455	−1.364	−2.490	0.013
	Turtle sightings	−116.464	−116.213	−0.282	−1.020	0.306
	Catches of sharks	−25.170	−24.610	1.439	0.640	0.522
Ligurian and Tyrrhenian Seas	Incidental catches of dolphins	−75.750	−63.700	−0.799	−3.850	0.000
	Incidental catches of turtles	−78.810	−74.668	−0.553	−2.650	0.008
	Dolphin sightings	−87.960	−87.816	−0.507	−5.500	0.000
	Whale sightings	−69.953	−64.972	−0.837	−2.630	0.009
	Turtle sightings	−83.605	−75.456	−0.950	−3.540	0.000
	Catches of sharks	−137.603	−116.773	−1.453	−5.090	0.000
North Adriatic Sea	Incidental catches of dolphins	−131.986	−131.881	−1.588	−4.640	0.000
	Incidental catches of turtles	−26.504	−26.504	−0.699	−1.370	0.171
	Dolphin sightings	−38.979	−33.339	−1.176	−2.710	0.007
	Turtle sightings	−30.308	−27.978	−0.697	−1.900	0.058
	Catches of sharks	−41.402	−28.689	−1.984	−3.210	0.001
Hellenic Seas	Incidental catches of dolphins	−18.050	−18.202	2.545	1.170	0.243
	Incidental catches of turtles	−7.573	−7.573	−0.021	−0.001	1.000
	Dolphin sightings	−26.246	−25.361	3.887	0.110	0.915
	Turtle sightings	−26.221	−24.840	−3.736	−0.100	0.923
	Catches of sharks	−20.262	−15.438	−10.469	−0.010	0.989

The null hypothesis tested is the absence of a time effect in the responses to questions on catches or sightings of large marine fauna in each Mediterranean case study. The full model (including time effect) was significant when the log-likelihood value was smaller than in the null model, and the coefficient of the time effect was significant at the 5% level (Z-test).

Intentional catches of dolphins for consumption have always been rare in the Mediterranean and only some fishers reported catching them for consumption: in Catalonia, 9% of the fishers in the early 1940s; in the Ligurian and Tyrrhenian Seas, 25% occasionally caught dolphins in the earlier periods. In general, fishers report that dolphin meat is not appreciated and preparing it is very time consuming compared to fish or other meat. Intentional culling of dolphins was encouraged by governments until the early 1970s and fishers interviewed in Italy and Greece confirmed that mass killings of dolphins were frequent in those times.

Incidental catches of turtles were relatively frequent only in the North Adriatic Sea, and were occasional or absent elsewhere (Figure 2). Only in the interviews of fishers of the Ligurian and Tyrrhenian Seas was there significant evidence of a decrease in the frequency of incidental catches of turtles and a decrease in the sightings of turtles (Table 2).

Whale sightings in the Ligurian and Tyrrhenian Seas, the only areas which provided responses that were amenable to statistical analysis, have become increasingly occasional and the change in observation frequencies is significant at the 5% level (Table 2, Figure 2).

**Figure 3:**
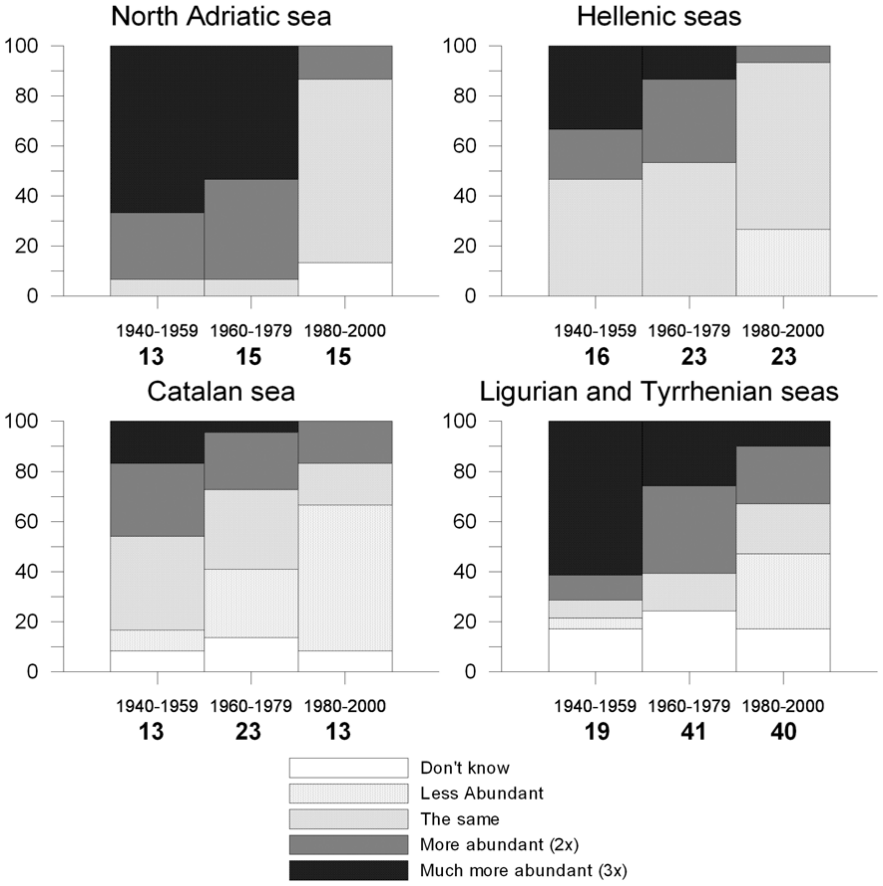
Trends in catches of cartilaginous fish. Frequencies in the responses to questions on catches of cartilaginous fish (sharks and rays) in each Mediterranean case study, by time period.

Shark catches in the four study areas have also decreased over time, with a higher frequency “more abundant” or “much more abundant” responses in the 1940–1959 and 1960–1979 study periods (Figure 3). These changes in frequencies were significant in the Italian study areas at the 5% level (Table 2). Among the responses in which the sharks in question could be identified to the genus or species level, interviewees in Catalonia declared that *Raja* spp., *Scyliorhinus canicula* and *Galeus melastomus* were and continue to be present in the catches. Conversely, and according to the interviews, three shark species, *Squalus acanthias*, *Prionace glauca* and *Cetorhinus maximus*, have never been more than occasional and do not show any particular trend. The sharks *Mustelus* spp. and *Squatina* spp. were cited to have been accidentally caught in the periods 1940–1959 and 1960–1979, but absent in the most recent period. 64% of fishers in Catalonia stated that smoothhounds, *Mustelus* spp., had disappeared from the catches before 1979. However, only 14% of the fishers were able to recognize angelsharks, *Squatina* spp., which suggests that they became practically extinct in the Catalan Sea much earlier, probably before 1959.

In the Ligurian and Tyrrhenian Seas, the interviewees declared that *Raja* spp. and *S. canicula* continue to be present in the catches, but dogfish *Squalus* spp. have become rare. *Mustelus* spp. and *Squatina* spp. were added to the dogfish as groups of species that have practically disappeared from trawl catches. The interviewees indicated the early 1980s as the time period in which angelsharks disappeared on the western Italian coasts (mid-1980s in Sardinia) and before 1990 for the disappearance of smoothhounds. The responses of both trawl and small scale fishers agree on the changes in abundance of shark species in the catches and on the time of disappearance of smoothhounds and angelsharks. The proportion of sharks in the trawl catches of the Ligurian and Tyrrhenian Seas decreased from 14.2% in the 1940–1959 period to 5.3% in the 1980–1999 period (Table 3).

**Table 3:** Proportion of cartilaginous fish in the catches of the Mediterranean trawl fleet in different periods.

	1940–1959	1960–1979	1980-present
Ligurian and Tyrrhenian Seas (trawl)	14.2% (10–40%)	10.3% (5–30%)	5.3% (5–10%)
North Adriatic Sea	12.7% (10–50%)	10.9% (5–30%)	5.8% (2–10%)
Hellenic Seas	11.4% (5–25%)	10.8% (0.1–25%)	9.3% (1–25%)

Proportion as % of catch in weight (average and min-max values between brackets) according to the interview survey results in case studies in which fishers could readily separate cartilaginous fish from bony fish.

In the Adriatic Sea, all shark species known to fishers, *Raja* spp., *Squalus* spp. and *Mustelus* spp, have become less abundant according to the interviews. Their abundance in the catches decreased by half, from 12.7% in the 1940–1959 period to 5.7% in the period 1980–1999 (Table 3). Another type of fish that used to be important in the catches of the North Adriatic is the sturgeon, *Acipenser* spp., which was last caught in 1966.

In the Hellenic Seas the general decrease in the abundance of sharks in the catches could not be attributed to any particular species, but the last catches of *Mustelus* spp. are reported in 1990. The species was reported to once have been very abundant outside all major river mouths.

The monk seal *Monachus monachus* is on the verge of extinction in the Mediterranean (and globally) and was extirpated in most of the study areas well before the Second World War [28], [31]. For this reason, only a few fishers were able to cite having seen or accidentally caught this species. In the Tuscan Archipelago (Tyrrhenian Sea) fishers noted its presence until the late 1970s, with occasional intentional catches until the early 1940s reported by small scale fishers. In the Aegean Sea, around 10% of fishers declared frequent or occasional sightings of seals, but these sightings were limited in the South Aegean. No living fisher from the North Adriatic has ever seen a monk seal.

**Figure 4:**
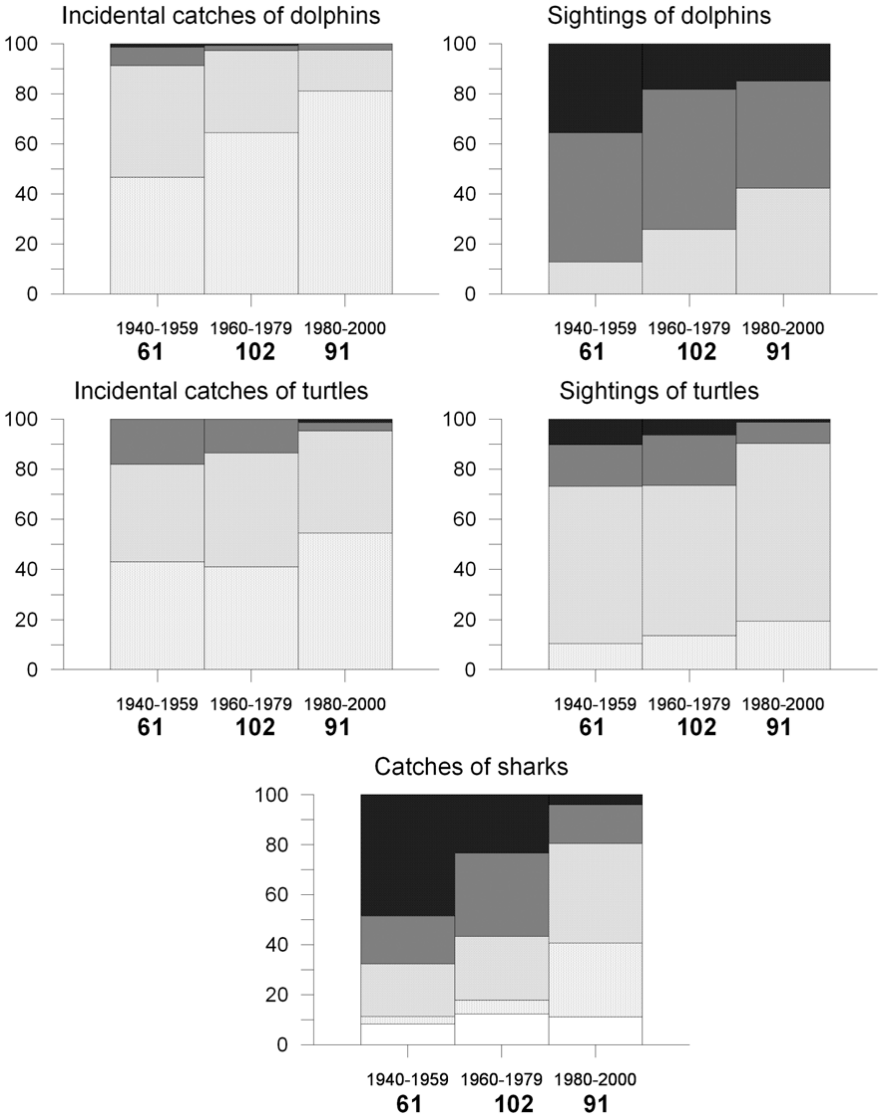
Trends in catches and sightings of large marine fauna, all areas combined. Frequencies in the responses to questions on catches or sightings of large marine fauna combining the different Mediterranean case studies, by time period.

When all the data for the different Mediterranean case studies (Figure 4 and Table 4) were combined, the trends became clearer, especially for dolphins and sharks. The proportion of interviewees that stated that dolphins were never caught incidentally in nets increased from *ca.* 50% to 80% in the 70 year period (Figure 4, top left). Moreover, dolphin sightings have also become less frequent (Figure 4, top right): in the period 1940–1959 less than 15% of the fishers declared that sightings were occasional, while currently 50% of the fishers state that sightings are only occasional. Both in the case of incidental catches and sightings the frequencies changed significantly over the study period at the 5% significance level.

**Table 4:** Results of the Generalized Linear Mixed Models on ordinal outcomes combining all cases studies.

Variable tested	Null model log-likelihood	Full model log-likelihood	coefficient of time effect	Z	p(Z)
Incidental catches of dolphins	−181.273	−163.049	−0.869	−5.250	0.000
Incidental catches of turtles	−182.313	−172.847	−0.732	−3.870	0.000
Dolphin sightings	−25.657	−233.694	−0.756	−5.720	0.000
Turtle sightings	−168.317	−156.088	−0.842	−4.230	0.000
Catches of sharks	−294.837	−252.938	−1.090	−7.820	0.000

The null hypothesis tested is that the responses to questions on catches or sightings of large marine fauna in the combined Mediterranean case studies do not change over time. The full model (including time effect) was significant when the log-likelihood value was smaller than in the null model, and the coefficient of the time effect was significant at the 5% level (Z-test).

Turtles were not frequently caught or sighted in the study period (Figure 4, middle panel); however, the proportion of fishers who reported frequent or very frequent catches or sightings decreased over time (both significant at the 5% level, Figure 4).

Shark catches were 2 or 3 times more abundant in the 1940–1959 study period than in the present, as declared by more than 60% of the fishers, and this proportion decreased significantly over time at the 5% significance level (Figure 4, bottom, and Table 4).

## Discussion

Our results show that the frequency of encounters between large marine fauna and Mediterranean fishermen in pilot study areas has decreased during the 70-year period 1940–2008: Mediterranean fishers at the beginning of the 21^st^ century accidentally caught or sighted less dolphins and turtles than in the mid 20^th^ century. The commercial catches of cartilaginous fish (“sharks”) have also decreased significantly. The abundance of other large marine fauna such as the monk seal or whales was already so low during the 20^th^ century that the interview survey did not produce sufficient data for a quantitative analysis. If we accept that commercial trawl fishers are independent observers of the marine system, these results suggest that the abundance of large marine fauna has decreased considerably during the 20^th^ century in the Mediterranean Sea (in agreement with the results of other studies; e.g. [25], [26]), and therefore fishers' observations during a lifetime of professional activity can provide a qualitative measure of this decline.

Fishing activities in the Mediterranean Sea have been exploiting marine coastal communities for centuries and the most vulnerable species disappeared long before the fishermen we interviewed started fishing. For example, [32] showed that the populations of hammerhead sharks had already started to decline in the Tyrrhenian Sea in the early 20th century, and that large predatory sharks in general have declined over the past 2 centuries. The decrease in chondrichthyans and large bony fish is also reported for the Adriatic Sea [26]. In the Mediterranean Sea, decreases in the landings and the number of elasmobranch species are well documented, including in the Gulf of Lions [33] and the northern Tyrrhenian Sea [32]. There are strong indications that the development of the bottom trawl fishery is directly related to this deterioration in the chondrichthyan population status [33], [34]. In other oceans, available long-term data series have revealed the impact of the fishing activity on elasmobranch populations, which is reflected in the reductions in species numbers and declining abundances [12], [35], [36]. In some cases, there is so little information on certain species [32] that trends cannot be assessed; however, it needs to be stressed that these same species, based on historical records, were once considered common [25]. It is therefore possible that although interviews with fishers or quantitative modelling cannot be used to evidence this, these species have effectively been eradicated.

Our results are in line with the declining trends reported by many authors for large marine species in the Mediterranean, particularly for dolphins [2], [3], [4]. Our objective was not so much to provide additional evidence confirming these declining trends or to quantitatively assess by-catches (as in [37]), but rather to evaluate whether fishers' memories can be reliably used as an information source which is complementary to scientific analysis ([17], [23]). Fishers have the advantage of observing the marine system on a daily basis, and they can provide valuable information when asked appropriate questions. Here we worked with retired fishers in order to obtain data for the longest time span possible, but we also acknowledge that precise data at a fine temporal resolution may be impossible to obtain due to imperfect memories. As a compromise we used 3 fixed time periods (1940–1959; 1960–1979 and 1980–1999 compared with the present) and qualitative questions which facilitated making comparisons (i.e. “the same”, “larger than”, etc.).

Given the lack of long time-series on the population abundances of marine organisms such as dolphins, turtles and sharks, fishers' perceptions could be a useful tool for reconstructing qualitative abundance indices ([23], [27], [38]). Furthermore, using traditional ecological knowledge as a complement to scientific research needs to be employed to its full potential in marine biological conservation ([23], [38], [39]). Enlisting fishers as long-time ecosystem observers is a methodology that complements other methodologies that have been used to reconstruct long-term trends in species abundance, such as using sales records, fishing logbooks or old naturalists' species descriptions [24], [26], [40], [41].

Evidence of the decline of the short-beaked common dolphin (*Delphinus delphis*) in the NW Mediterranean, which began in the early 1970s, was summarized by [2]. Later [3] confirmed that the trend was general throughout the Mediterranean Sea. Although this species used to be common in the entire Mediterranean Sea well into the 20^th^ century [3], it is now only common in the Alboran Sea and in parts of the Aegean, with much reduced populations elsewhere in the Mediterranean. However, [42] cite that it is regularly seen in many areas of the Greek Seas and its distribution here shows a completely different situation compared to the rest of the Mediterranean. Therefore, the Greek Seas seem to host an important pool of the Mediterranean short-beaked common dolphin, in addition to the north Alboran Sea [3], [43].

Although there is no comparably detailed information on the other two dolphin species, different studies point in the same direction [4], [9], [44]. As anecdotal evidence, several Catalan fishers interviewed stated that in the 1940s and 1950s large pods of hundreds of individuals were commonly seen, both close to the shore and offshore, while nowadays the pods are composed of several individuals only.

Our results concerning sensitive demersal sharks and sturgeons also provided an *ante quem* date for their local extinction in different areas: smoothhounds *Mustelus* spp. are likely to have disappeared in the Catalan Sea before 1979, and angelsharks *Squatina* spp. before 1959. In western Italy, angelsharks would have disappeared by the early 1980s near the mainland and the mid-1980s in Sardinia. Smoothhounds became functionally extinct in 1990 in Italy and Greece, with only sporadic records thereafter. The sturgeon *Acipenser* spp. had become extinct in the North Adriatic by 1966.

The monk seal (*Monachus monachus*) was an abundant marine mammal in the Mediterranean until Roman times [31]. The reasons for the recent population decline which has led to the species status of critically endangered include increased human pressure that displaces seals from their habitat, destruction of caves used for hauling out and breeding, continued mortality due to being caught as by-catch by fisheries, deliberate aggression by fishermen to eliminate a competitor even in countries and areas where the species is legally protected, disease, pollution and impoverished genetic diversity [45]. According to [31] it was already heavily exploited for different human uses in ancient times, and its distribution range had already diminished considerably. Currently it is only present on the Hellenic and Turkish coasts, as well as locally in northern Africa [28]. Consequently the scarce information that was obtained in our survey refers only to sporadic contacts with an already relict population.

The causes for the decline in the abundance of large marine fauna in the Mediterranean are probably a combination of direct human impacts that have grown in intensity in the 20^th^ century and the intrinsic characteristics of this fauna, such as slow growth rates, high longevity (e.g. 20 years in *Etmopterus spinax*: [46]), low fecundity and high trophic position [13] [16] [32]. The impact of indirect human vectors of change might have played an additional role, but this is difficult to assess. For instance, [41] documented that the local extinction of a deep-water shrimp *Aristaeomorpha foliacea* was probably due to the warming and salinization of Mediterranean deep waters due to the decreased freshwater flow from the Nile since the completion of the High Aswan Dam in 1964.

As discussed by [21], few present and future fishers (as well as the general population) in the Mediterranean will be in a position to appreciate the steep decline in the abundance of large marine species during the 20^th^ century due to the rapidly shifting baselines [47].
